# Migraine day frequency in migraine prevention: longitudinal modelling approaches

**DOI:** 10.1186/s12874-019-0664-5

**Published:** 2019-01-23

**Authors:** Gian Luca Di Tanna, Joshua K. Porter, Richard B. Lipton, Alan Brennan, Stephen Palmer, Anthony J. Hatswell, Sandhya Sapra, Guillermo Villa

**Affiliations:** 1 Economic Modelling Center of Excellence, Amgen Europe GmbH, Suurstoffi 22, P.O. Box 94, CH-6343 Rotkreuz, Switzerland; 2 Albert Einstein College of Medicine, New York, NY 10461 USA; 3 ScHARR, University of Sheffield, Sheffield, UK; 4 Centre for Health Economics, University of York, York, UK; 5 Delta Hat Limited, Nottingham, UK; 6 Amgen Inc., Thousand Oaks, CA 91320 USA

**Keywords:** Erenumab, Migraine, Migraine frequency, Modelling, Negative binomial, Beta-binomial

## Abstract

**Background:**

Health economic models are critical tools to inform reimbursement agencies on health care interventions. Many clinical trials report outcomes using the frequency of an event over a set period of time, for example, the primary efficacy outcome in most clinical trials of migraine prevention is mean change in the frequency of migraine days (MDs) per 28 days (monthly MDs [MMD]) relative to baseline for active treatment versus placebo. Using these cohort-level endpoints in economic models, accounting for variation among patients is challenging. In this analysis, parametric models of change in MMD for migraine preventives were assessed using data from erenumab clinical studies.

**Methods:**

MMD observations from the double-blind phases of two studies of erenumab were used: one in episodic migraine (EM) (NCT02456740) and one in chronic migraine (CM) (NCT02066415). For each trial, two longitudinal regression models were fitted: negative binomial and beta binomial. For a thorough comparison we also present the fitting from the standard multilevel Poisson and the zero inflated negative binomial.

**Results:**

Using the erenumab study data, both the negative binomial and beta-binomial models provided unbiased estimates relative to observed trial data with well-fitting distribution at various time points.

**Conclusions:**

This proposed methodology, which has not been previously applied in migraine, has shown that these models may be suitable for estimating MMD frequency. Modelling MMD using negative binomial and beta-binomial distributions can be advantageous because these models can capture intra- and inter-patient variability so that trial observations can be modelled parametrically for the purposes of economic evaluation of migraine prevention. Such models have implications for use in a wide range of disease areas when assessing repeated measured utility values.

**Electronic supplementary material:**

The online version of this article (10.1186/s12874-019-0664-5) contains supplementary material, which is available to authorized users.

## Background

Migraine is a common neurological disorder characterised by debilitating, recurrent headaches, often divided into episodic (EM) and chronic (CM) forms based on month headache days (MHD) and monthly migraine days (MMD) (EM, 4–14 MMD and < 15 MHD, or CM, ≥15 MHD and ≥ 8 MMD) [1–3]. Migraine pain is typically unilateral, pulsating in quality, of moderate or severe intensity and aggravated by routine physical activity, such as walking or climbing stairs. In addition, diagnosis depends on the presence of associated symptoms of nausea, vomiting, photophobia or phonophobia in various combinations [1, 2, 4]. The burden of migraine is considerable, both in terms of the physical and emotional effects on the individual, and the economic impact of lost productivity and healthcare resource use [5]. It is ranked as the leading cause of neurological disability worldwide and is one of the five leading causes of long-term disability [6, 7].

Preventive treatment intended to reduce the frequency and severity of headaches is an important aspect of management; all patients with CM would benefit from preventative treatment. Among patients with EM, experiencing 4 or more headache days per month is a leading reason for considering preventative therapy [8]. MMD and MHD are counts that have values that include zero as well as positive integers; count data typically have skewed distributions [9]. Reductions in the frequencies of migraine days (MDs) and headache days are key measures of the efficacy of migraine prophylaxis.

Clinical studies typically examine the mean change in MMD frequency; patient-level data are not widely published. However, examining the mean change in MMD frequency across a cohort of patients may not capture the clinically meaningful effects of migraine prevention, such as the improvement in an individual’s ability to perform daily activities or health-related quality of life. Furthermore, examining the mean change in the MMD frequency for a population in clinical studies may not be applicable in the real-world, as treatments may shift the frequency distribution.

A higher frequency of MMD per 28 days is associated with lower health-related quality of life, increased use of medical resources, acute medication use and increased productivity losses, with the impact of each additional MD increasing with overall frequency. As such, the average outcomes across a patient cohort may not be the same as the outcomes of a patient with the average MMD frequency. The frequency distribution of MHD and MMD is important when it comes to modelling the effectiveness and cost-effectiveness of prophylaxis [10]. Previous analyses examining cost-effectiveness models for migraine have approached this issue by defining health states as categorical event frequency (transition from ≥15 MHD to < 15 MHD) or as response status (≥50% reduction in MHD) [10–13] which may not adequately account for inter-patient variability. These models group together a heterogeneous set of patient outcomes, rendering the models less precise; for example Markov models tend to categorise patients into broad categories, which can be challenging when assessing benefits. In general, categorising count/continuous variables can lead to several problems including loss of information and may also increase the risk of false positives [14]. Furthermore, use of a data-derived ‘optimal’ cutpoint may lead to bias [15]. Migraine is a disease with considerable variability in the frequency, duration and severity of migraine attacks [16]. Therefore, there is a need for an approach that estimates the change in mean frequency of MMD but also the distribution of individual patients by MMD frequency within a cohort at subsequent time points.

Selection of the most appropriate model is important when fitting MHD or MMD data [17]. There are several approaches to modelling these data. Reports on modelling MMD frequency in the literature are limited but previous analyses have used Poisson and negative binomial to model headache day frequency [17–21]. Zero-inflated variants of these distributions have also been used to improve goodness-of-fit [17, 22]. The Poisson distribution belongs to the family of discrete probability distributions traditionally used to model count data. In general, the model assumes that the mean and variance of the count data are equal [23]. It is considered appropriate for unrestricted count data [24], and because MMD frequency is a count variable, Poisson distribution may be considered an eligible model. However, its ability to model the variation seen in the patient-level data has proved limited [20, 25] due to insufficient accounting for overdispersion (where a single parameter is insufficient to characterise the mean and variance) [26]. More recently, thanks to Shmueli et al. there has been a resurgence in interest in Conway-Maxwell-Poisson distributions, originally proposed by Conway and Maxwell to handle queuing systems [27, 28]. The main characteristic of these distributions, which are an extension of the Poisson distribution, is the ability to handle both overdispersion and underdispersion. These distributions are limited, however, due to the lack of a hierarchical model to assess repeated measurements and parameterization that is not made directly via the mean of counts, making these distributions not easily comparable to other count regression models. By contrast, the negative binomial distribution, which uses an additional dispersion parameter to represent the additional variation seen in the data, has provided superior fits when modelling migraine populations [17, 20].

A preliminary analysis, based on cross-sections of the data, has indicated that the beta-binomial is an alternative distribution that could be used to model MMD frequency data and has been shown to provide comparable fits to the negative binomial models [25]. The beta-binomial model is commonly used to account for intraclass correlation coefficients (ICC) among dichotomous outcomes in cluster sampling [29]. The use of the beta-binomial model may offer some advantages because the outcome can be restricted to a maximum number of possible successes (i.e. a maximum of 28 MMD per 4-week period).

In order to assess the feasibility of fitting MMD data using negative binomial or beta-binomial models, longitudinal data from two erenumab studies were examined [30, 31]. Erenumab is a fully human monoclonal antibody that specifically binds to and blocks the calcitonin gene-related peptide (CGRP) receptor [32]. Erenumab has been evaluated as a prophylactic treatment for migraine in 2 pivotal clinical trials in patients with EM and CM [30, 31, 33].

To the best of our knowledge, longitudinal negative binomial and beta-binomial regression models that accommodate over-dispersed data have not been used previously in the assessment of MMD frequency. Here, we describe an assessment of these models of the change in MMD frequency, using data from the placebo and erenumab 140 mg arms of two pivotal erenumab clinical trials.

## Methods

### Models specification

Three longitudinal regression models were evaluated for their ability to estimate the frequency distribution of MMD: multilevel/hierarchical negative binomial regression (with constant dispersion parameter over time), multilevel beta-binomial regression (with constant ICC over time) and the multilevel Poisson model. The distributions in the erenumab cohorts of the studies were estimated and compared to the observed distribution across the double-blind period. Zero-inflated negative binomial models with robust standard errors clustering at patient level (presented in Additional file 1: Table S1 and Additional file 2: Figure S1) were also fitted, but only the non-zero-inflated were considered here because zero-inflated models did not improve the model’s fit, and there was no substantial inflation of zeros due to the eligibility criteria for the study [20].

### Negative binomial distribution

The negative binomial distribution is an extension of the Poisson distribution and includes a dispersion parameter (***τ***) to account for overdispersion in the data. The negative binomial does not have an upper bound (unlike the beta binomial), so it is possible for high mean frequencies to result in predictions above the maximum of 28 MDs per month. The dispersion parameter is estimated based on the mean MD frequency.

The negative binomial probability function [34] is defined as:

1 $$ {\displaystyle \begin{array}{c}P\left(Y=k\right)=\frac{T\left(k+\tau \right)}{k!T\left(\tau \right)}{\left(\frac{\uptau}{\uptau +\uplambda}\right)}^{\tau }{\left(\frac{\uplambda}{\uplambda +\uptau}\right)}^k\\ {}k=0,1,2,\dots \end{array}} $$

Where:

- *P(Y = k*) is the probability of a patient experiencing *k* MDs per 28 days
- *λ* is the mean MDs per 28 days
- *τ* is the dispersion parameter.

Note that the dispersion parameter estimated by Stata is 1 divided by the dispersion parameter in eq. 1. To ensure that the function sums to unity, the model divides each estimated frequency by the cumulative frequency of the negative binomial at *Y* = 28 (28 MDs per 28 days).

This regression framework can accommodate differences in MMD frequency and the variation in frequency between patients at different time points. Parameters that accommodate overdispersion were estimated for the negative binomial regression, referred to as the dispersion parameter.

### Beta-binomial distribution

The beta-binomial data model is a combined model of the beta and binomial distributions. It is used to model the number of successes (counts) over a number of binomial trials, when the probability of success is a beta distribution with two specific parameters (*α* and *β*) [35]. In general, the beta-binomial distribution accounts for the fact that the observed events are not equally distributed across patients and can be used to assess non-linear associations [35]. In the beta-binomial distribution, the count data at each observation timepoint are regarded as a set of 28 binary outcomes (MD or non-MD) grouped by patient. The *α* and *β* parameters of the beta-binomial distribution can be calculated from the mean and ICC, which represents the strength of the correlation between days for the same patient, i.e. daily outcomes are likely to be similar for the same patient.

The beta-binomial probability function is specified as follows:

2 $$ {\displaystyle \begin{array}{c}P\left(Y=k\right)=\left(\begin{array}{c}N\\ {}k\end{array}\right)\frac{B\left(\alpha +k,\beta +N-k\right)}{B\left(\alpha, \beta \right)}\\ {}k=0,1,2,\dots \end{array}} $$

Where:

- *k* is the number of MDs
- *P* (Y = k) is the probability of patients experiencing *τ* MDs
- *N* is the number of days in the cycle (28 days)
- *B* () is the beta function
- *α* and *β* are the parameters of the underlying beta distribution.

The ICC is assumed constant over time and equal to 1 / (1 + *α* + *β*).

### Erenumab clinical trial data

Table 1 summarises some of the key characteristics of the patients from the two erenumab studies. The patient data used in the modelling analysis were taken from two pivotal clinical trials of erenumab as migraine prophylaxis; one in patients with EM (NCT02456740) [30], the other in patients with CM (NCT02066415) [31]. Patients enrolled in the EM study had 4 to 14 MDs and fewer than 15 headache days per month (28 days) at baseline. Patients in the CM study had 15 or more headache days per month at baseline, of which at least 8 were MDs. Both of these randomised, double-blind studies compared erenumab 70 mg and 140 mg, administered every 28 days by subcutaneous injection, with placebo [30, 31]. Patients received double-blind treatment for 12 weeks (CM study) or 24 weeks (EM study), and efficacy was assessed as the change in mean MMD from baseline. This analysis focuses on the erenumab 140 mg dose only. Patient-level data were obtained for the patients in each study, with the following variables extracted for use in the analysis: subject ID, MMD frequency, visit, and treatment. This approach allows the regression models to estimate both the change in MMD frequency over time and the dispersion parameters required to reproduce the distribution of patient-level MMD frequency.

**Table 1:** Baseline characteristics of patients in the erenumab clinical trials [31, 43]

Characteristic	Episodic migraine^a^ (NCT02456740)		Chronic migraine^b^ (NCT02066415)	
Group	Placebo	Erenumab 140 mg	Placebo	Erenumab 140 mg
Number of patients	319	319	286	190
Mean age (SD)	41.3 (11.2)	40.4 (11.1)	42.1 (11.3)	42.9 (11.1)
Sex, n (%)				
Male	45 (14.1)	47 (14.7)	60 (21.0)	30 (15.8)
Female	274 (85.9)	272 (85.3)	226 (79.0)	160 (84.2)
Race, n (%)				
White	277 (86.8)	293 (91.8)	268 (93.7)	184 (96.8)
Black	24 (7.5)	18 (5.6)	11 (3.8)	6 (3.2)
Other	18 (5.6)	8 (2.5)	7 (2.4)	0 (0.0)
Baseline MMD	8.2 (± 2.5)	8.3 (± 2.5)	18.2 (± 4.7)	17.8 (± 4.7)

*n* number, *MMD* monthly migraine days, *SD* standard deviation

^a^Defined as patients experiencing 4–14 headache days per 28 days, 4–14 migraine days per 28 days

^b^Defined as patients experiencing ≥15 headache days per 28 days, ≥8 migraine days per 28 days

Goodness of fit of the regression models was assessed by estimating the root mean squared errors (RMSE) across the estimated values compared with trial observations, mean absolute errors (MAE) and visual inspection of the predicted distributions. The models could not be compared via Akaike’s information criteria (AIC) or Bayesian information criterion (BIC) because the beta-binomial model was performed on the augmented dataset.

Predicted MMD values and 95% confidence intervals were calculated with the Delta method. For economic modelling purposes, the mean MMD frequencies were extrapolated beyond the trial observation, up to a maximum of 2 years, after which no further change in MMD frequency was assumed. The models tested were exponential, logistic, log-logistic and Gompertz. Extrapolation was performed using a logistic function, the best-fitting function out of the models tested. Further information on the extrapolations can be found in the supplementary material (Additional file 3: Figure S2).

All analyses were performed using Stata Statistical Software: Release 15.0 (StataCorp LLC, College Station, TX, USA) [36], and the Stata codes to fit the regression models proposed are located in the Additional file 4: Technical appendix.

## Results

### Patient characteristics

Baseline patient characteristics were similar in the two studies, despite differences in MMD frequency at baseline (patients experienced an average of 8 vs 18 MDs per 28 days for EM and CM respectively) (Table 1). In the EM study, mean age was 40.9 years, and the majority of patients were white (89.1%) and female (85.2%). In the CM study, these figures were similar with a mean age of 42.5 years, 94.2% were white and 82.8% were female.

### MMD frequency modelling

**Fig. 1:**
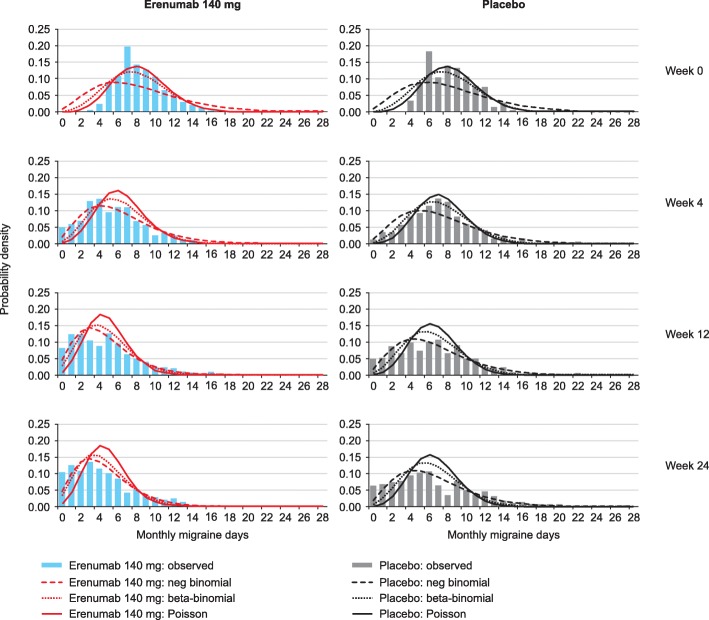
Estimated and actual MMD distributions in the EM study at weeks 0, 4, 12 and 24

**Fig. 2:**
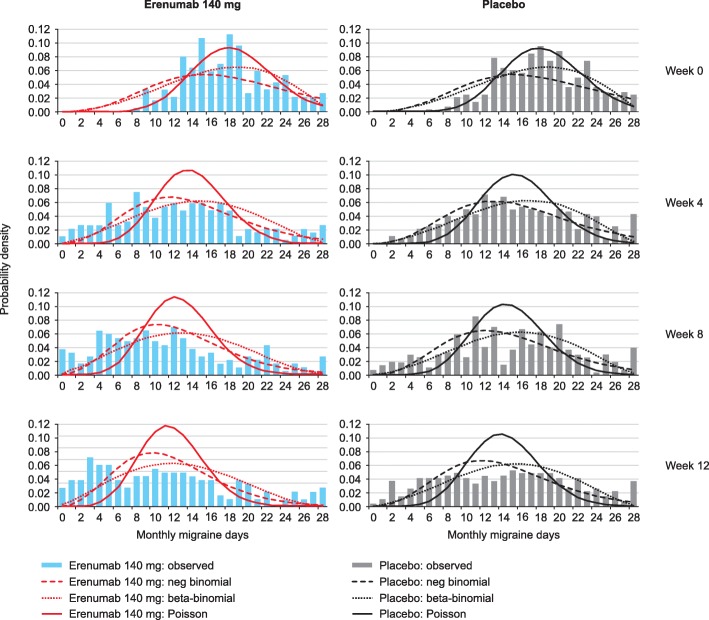
Estimated and actual MMD distributions in the CM study at weeks 0, 4, 8 and 12

Figure 1 shows the predicted distributions of patients in the two cohorts compared with the study observations at weeks 0, 4, 12 and 24 in the EM study. In the EM study, the predicted distributions for both regression models show a good fit with the actual data at 4, 12 and 24 weeks (Fig. 1). Figure 2 shows the predicted distributions and actual data at weeks 0, 4, 8 and 12 in the CM study. The predicted distributions show a good fit to the actual observations in the EM and CM study; the RMSE estimates were 0.075 and 0.082 for negative binomial regression, 0.102 and 0.081 for beta-binomial regression and 0.142 and 0.152 for Poisson regression for EM and CM studies respectively. The MAE estimates were 0.246 and 0.330 for negative binomial regression, 0.336 and 0.339 for beta-binomial regression, and 0.466 and 0.654 for Poisson regression for EM and CM studies respectively. For negative binomial regression, the dispersion parameter was 0.2397 for the EM study and 0.1323 for the CM study; for the beta-binomial regression, the ICC values were 0.0297 and 0.1370 for the EM and CM studies (Tables 2 and 3).

**Table 2:** EM regression output for negative-binomial, beta-binomial and Poisson

Negative binomial (Dispersion parameter 0.2397)						Beta-binomial (ICC 0.0297)						Poisson					
Covariate	Predicted MD Frequency^a^	95% CI	FRR	95% CI	*P* value		Predicted MD Frequency^a^	95% CI	Coefficient	95% CI	*P* value		Predicted MD Frequency^a^	95% CI	FRR	95% CI	*P* value
Week 0	8.261	(7.622, 8.900)	–	–	–	Week 0	7.944	(7.334, 8.555)	–	–	–	Week 0	8.333	(7.790, 8.877)	–	–	–
Week 4	7.199	(6.714, 7.684)	0.746	(0.712, 0.782)	< 0.001	Week 4	7.081	(6.595, 7.567)	−0.292	(− 0.345, -0.239)	< 0.001	Week 4	7.312	(6.824, 7.800)	0.768	(0.737, 0.800)	< 0.001
Week 8	6.731	(6.278, 7.185)	0.693	(0.661, 0.727)	< 0.001	Week 8	6.672	(6.215, 7.128)	−0.379	(−0.433, -0.324)	< 0.001	Week 8	6.847	(6.385, 7.310)	0.718	(0.688, 0.749)	< 0.001
Week 12	6.4337	(6.005, 6.862)	0.651	(0.620, 0.683)	< 0.001	Week 12	6.386	(5.952, 6.820)	−0.447	(−0.503, -0.391)	< 0.001	Week 12	6.555	(6.108, 7.002)	0.677	(0.649, 0.706)	< 0.001
Week 24	6.421	(6.002, 6.840)	0.634	(0.604, 0.666)	< 0.001	Week 24	6.293	(5.866, 6.720)	−0.486	(−0.542, -0.429)	< 0.001	Week 24	6.552	(6.105, 6.998)	0.662	(0.634, 0.690)	< 0.001
Treatment (Erenumab vs Placebo)			0.761	(0.702, 0.825)	< 0.001	Treatment (Erenumab vs Placebo)			−0.327	(−0.362, -0.291)	< 0.001	Treatment (Erenumab vs Placebo)			0.764	(0.705, 0.827)	< 0.001
RMSE		0.075				RMSE		0.102				RMSE		0.142			
MAE		0.246				MAE		0.336				MAE		0.466			

Regression output analysis was based on the whole sample of patients (4438 observations)

*CI* confidence interval, *FRR* frequency rate ratio, *ICC* intraclass correlation coefficient, *MAE* mean absolute error, *MMD* monthly migraine day, *RMSE* root mean squared error

^a^In the placebo arm

**Table 3:** CM regression output for negative binomial, beta-binomial and Poisson

Negative binomial (Dispersion parameter 0.1323)						Beta-binomial (ICC 0.1370)						Poisson					
	Predicted MD Frequency^a^	95% CI	FRR	95% CI	*P* value		Predicted MD Frequency^a^	95% CI	Coefficient	95% CI	*P* value		Predicted MD Frequency^a^	95% CI	FRR	95% CI	*P* value
Week 0	18.111	17.052, 19.171)	–	–	–	Week 0	17.111	(16.156, 18.066)	–	–	–	Week 0	18.298	(17.373, 19.223)	–	–	–
Week 4	15.418	(14.579, 16.257)	0.783	(0.754, 0.812)	< 0.001	Week 4	15.843	(15.028, 16.657)	−0.256	(− 0.321, -0.192)	< 0.001	Week 4	15.577	(14.770, 16.385)	0.798	(0.773, 0.824)	< 0.001
Week 8	14.538	(13.759, 15.317)	0.721	(0.694, 0.749)	< 0.001	Week 8	15.256	(14.484, 16.027)	−0.359	(−0.426, -0.293)	< 0.001	Week 8	14.688	13.919, 15.457)	0.739	(0.715, 0.764)	< 0.001
Week 12	13.997	(13.242, 14.753)	0.696	(0.670, 0.724)	< 0.001	Week 12	14.894	(14.146, 15.641)	−0.408	(−0.475, -0.341)	< 0.001	Week 12	14.142	13.397, 14.887)	0.715	(0.692, 0.739)	< 0.001
Treatment (Erenumab vs Placebo)			0.828	(0.767, 0.894)	< 0.001	Treatment (Erenumab vs Placebo)			−0.3600	(−0.430, -0.290)	< 0.001	Treatment (Erenumab vs Placebo)			0.831	(0.770, 0.896)	< 0.001
RSME		0.082				RMSE		0.081				RMSE		0.152			
MAE		0.330				MAE		0.339				MAE		0.654			

Regression output analysis was based on the whole sample of patients (1872 observations)

*CI* confidence interval, *FRR* frequency rate ratio, *ICC* intraclass correlation coefficients, *MAE* mean absolute error, *MMD* monthly migraine day, *RMSE* root mean squared errors

^a^ In the placebo arm

For the EM study, the negative binomial mean MMD for weeks 0, 4, 12 and 24 were 8.261, 7.199, 6.434 and 6.421, respectively. For beta-binomial regression, the mean MMD for weeks 0, 4, 12 and 24 were 7.945, 7.080, 6.386 and 6.293, respectively (Table 2). For the CM study, the negative binomial mean MMD for weeks 0, 4, 8 and 12 were 18.111, 15.418, 14.538 and 13.997, respectively. For beta-binomial regression the mean MMD for weeks 0, 4, 8 and 12 were 17.111, 15.843, 15.256 and 14.894, respectively (Table 3).

**Fig. 3:**
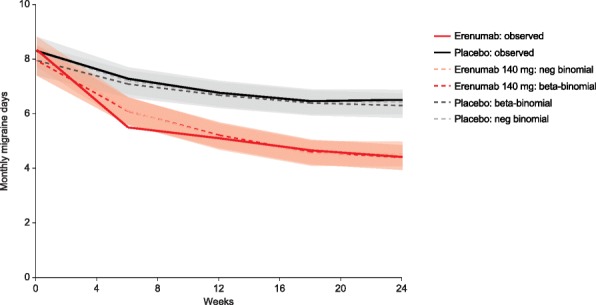
MMDs over 24 weeks of the EM study: negative binomial and beta-binomial longitudinal regression estimates and observed data. neg, negative. 95% confidence intervals for the negative and beta-binomials indicated by the shaded grey (placebo) and red (erenumab)

**Fig. 4:**
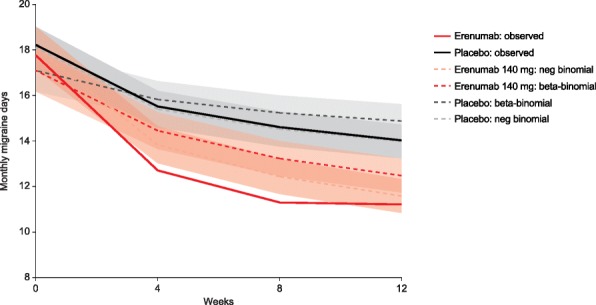
MMDs over 12 weeks of the CM study: negative binomial and beta-binomial longitudinal regression estimates and observed data. neg, negative. 95% confidence intervals for the negative and beta-binomials indicated by the shaded grey (placebo) and red (erenumab)

The predicted distributions observed in the CM study appeared a less close fit than for the EM study, which reflects the greater level of variability in the data from the CM study. Figure 3 presents the mean MMD frequencies for placebo predicted by the negative binomial and beta-binomial regression models compared with the observed mean values from patients with EM over the 24-week study period. Figure 4 presents the equivalent data for patients from the 12-week study in CM. In both studies, the modelled data from the two regressions show a closer fit with the observed values, compared with the Poisson reference model.

## Discussion

This analysis is an assessment of the ability of longitudinal parametric models to capture intra- and inter-patient variability in MMD frequency over time, using data from two erenumab clinical trials as examples. Patients with migraine experience considerable day-to-day variability in the frequency, duration and severity of attacks [16]. This approach was used to estimate patient distribution accurately by the frequency of MMD using mean MMD values for the overall patient population. Modelling MMD with negative binomial and beta-binomial longitudinal regression models can be advantageous because they can accommodate overdispersed data (with a variance larger than the mean) and account for the variation in MMD both within and between individual patients.

The approaches described here allows the distribution of individual patients by MMD to be modelled using only the clinical endpoint of the studies - the mean change from baseline in MMD compared with placebo at a single time point. The beta-binomial regression method allows restriction of the maximum successes (i.e. maximum of 28 MDs), whereas the negative binomial does not. Despite this, the negative binomial showed a better goodness of fit to the MD distributions than beta-binomial. The modelled data from the negative and beta-binomial regressions show a closer fit with the observed values, compared with the Poisson reference model. The zero-inflated negative binomial regressions did not substantially improve the goodness of fit of the predicted distributions. In contrast to clinical trial populations that may have a lower bound of MDs per month, the zero-inflation model may be more useful in a real-world population where a greater proportion of people have zero MDs in a month.

The choice of distributions is important when measuring count data. The Poisson and negative binomial distributions have been used in previous studies to model count data [20, 23, 37] and have also been used to approximate headache day frequency data in published migraine studies [17, 38]. However, these distributions may be inappropriate when event counts are limited by a maximum possible frequency or measuring multimodal distributions. The Poisson and negative binomial distribution have indefinite support for positive integers and, therefore, have the potential to generate inappropriate values, especially with migraine cohorts of higher MD frequency.

Modelling data as continuous events rather than categorising data has many advantages, including the reduction of bias and more accurately estimating the extent of variation in outcomes between groups [14].This analysis takes the approach of modelling migraine frequency as a continuous outcome and addresses a key limitation of previous modelling approaches which define health states by categorical event frequency or response status. The proposed approach also provides a greater capability to model indirect comparisons than previous models, as the published endpoints of clinical studies (i.e. mean change in MMD) can be used to estimate the distributions of patients, assuming the patient-level variation is similar across cohorts. Using a count-based structure makes indirect comparisons straightforward because data can be linked to study primary endpoints. Estimating the distribution of patients by MMD also allows outcomes linked to MD frequency (such as health-related quality of life or pain medication use) to be quantified directly as a function of frequency. Furthermore, because clinical trials in migraine are commonly placebo-controlled, this approach could be used to parameterise indirect comparison in migraine prevention where patient-level frequency data are not available.

While this approach addresses key limitations of previous approaches, such as defining health states by categorical event frequency or response status, some potential improvements could be made to it. The implementation of a negative binomial regression with upper bound (28 MDs) could be considered and treatment-visit interactions could be included. Additionally, the data are required to fit to the smooth distributions of the model; however, this is not always the case. The predicted distributions observed in the CM study did not fit as well as the EM study owing to the greater spread in distribution in the CM study and may also be due to the differences in the patient populations between the EM and CM cohorts. Therefore, alternative approaches may be required to better model these cohorts.

The method described here has applications in economic evaluations of preventative medication and policy decisions in migraine. The parametric approach proposed can be used to perform extrapolations of treatment effects beyond trial observations. Extrapolation of data is particularly relevant when considering economic evaluations [39] as patient-level data collected within the duration of clinical studies are often too short to assess the long-term relationship between migraine frequency and health status. Further research may consider how such data should be extrapolated into the future, as whilst survival-modelled extrapolation has become well-established and standardised, the parametric approach is relatively novel, and the way in which the data can be best extrapolated is yet to be defined [40]. Furthermore, there is an inherent risk to extrapolation, as the clinical trajectory can be uncertain.

Modelling outcomes as continuous variables rather than health states has advantages when data are limited. Therefore, this approach has implications for use in various disease analyses which have simplified continuous outcomes associated with health states, which may result in loss of information or bias. This approach could be used to evaluate the disease progression of patients with HIV/AIDS, where multistate Markov models based on CD4 cell counts have previously been used [41] or modelling health assessment questionnaire (HAQ) scores in patients with psoriatic arthritis [42].

## Conclusions

Modelling MMD with regression models that can accommodate overdispersion in a longitudinal framework is a statistically valid method to estimate the variation in MMD, both within and between individual patients. This approach, which estimates the distribution of patients by MMD, allows outcomes (such as health-related quality of life or pain medication use) to be directly quantified and linked to MD frequency. This has important applications in the evaluation of preventive medications for migraine and beyond.

## Additional files

Additional file 1: **Table S1.** EM and CM regression output for zero-inflated negative binomial. Contains the data for the EM and CM regression output for the zero-inflated negative binomial model. (DOCX 17 kb)

Additional file 2: **Figure S1.** Placebo modelling data: MMDs of the EM study (a) and the CM study (b) negative binomial regression, beta binomial regression and zero-inflated negative binomial regression. Contains placebo modelling data of MMDs of the EM and CM study for the regression models. (EPS 2448 kb)

Additional file 3: **Figure S2.** Mean MD extrapolations based on regression model predicted means. a) and b) EM regression models c) and d) CM regression models. Parametric extrapolations of mean MMD are based on the predicted means produced by the beta-binomial and negative binomial regression models of both EM and CM. The mean MMD frequency plateaued after approximately 6 months (24 weeks). Fitted values refer to the logistic, exponential and Gompertz functions. (EPS 2384 kb)

Additional file 4: Implementation of regression models in Stata. The technical appendix contains the Stata codes which were used to fit the regression models. (DOCX 13 kb)

## Abbreviations

AIC: Akaike’s information criteria
AIDS: Acquired immunodeficiency syndrome
BIC: Bayesian information criterion
CGRP: Calcitonin gene related peptide
CM: Chronic migraine
EM: Episodic migraine
HIV: Human immunodeficiency virus
ICC: Intraclass correlation coefficients
MAE: Mean absolute error
MHD: Monthly headache days
MMD: Monthly migraine days
RMSE: Root mean squared errors
