# Supplementary material for: Migraine day frequency in migraine prevention: longitudinal modelling approaches
Source: BMC Med Res Methodol. 2019 Jan 23;19:20. doi: 10.1186/s12874-019-0664-5 (PMC6343253; doi:10.1186/s12874-019-0664-5)
Supplement: Supplementary file 1 — Table S1. EM and CM regression output for zero-inflated negative binomial. Contains the data for the EM and CM regression output for the zero-inflated negative binomial model. (DOCX 17 kb) [file 12874_2019_664_MOESM1_ESM.docx]

**Table S1** EM and CM regression output for zero-inflated negative binomial

| **EM** | | | | | | **CM** | | | | | |
| --- | --- | --- | --- | --- | --- | --- | --- | --- | --- | --- | --- |
| **Zero-inflated negative binomial** (Dispersion parameter: 0.1466) | | | | | | **Zero-inflated negative binomial** (Dispersion parameter: 0.127) | | | | | |
| **Covariate** | **Predicted MD Frequency*** | **95% CI** | **Coefficient** | **95% CI** | **P value** | **Covariate** | **Predicted MD Frequency*** | **95% CI** | **Coefficient** | **95% CI** | **P value** |
| **Week 0** | 8.267 | (7.878, 8.656) | - | - | - | **Week 0** | 18.238 | (17.508, 18.969) | - | - | - |
| **Week 4** | 7.258 | (6.886, 7.630) | -0.241 | (-0.282,  -0.201) | <0.001 | **Week 4** | 15.527 | (14.820, 16.234) | -0.226 | (-0.2595,  -0.1927) | <0.001 |
| **Week 8** | 6.799 | (6.423, 7.175) | -0.286 | (-0.333,  -0.238) | <0.001 | **Week 8** | 14.641 | (13.919, 15.362) | -0.289 | (-0.3288,  -0.2493) | <0.001 |
| **Week 12** | 6.509 | (-6.135, 6.884) | -0.332 | (-0.381,  -0.284) | <0.001 | **Week 12** | 14.096 | (13.337, 14.855) | -0.329 | (-0.3742,  -0.2828) | <0.001 |
| **Week 24** | 6.506 | (6.110, 6.903) | -0.335 | (-0.388,  -0.282) | <0.001 |  |  |  |  |  |  |
| **Treatment (Erenumab vs Placebo)** | | | -0.214 | (-0.284,  -0.145) | <0.001 | **Treatment (Erenumab vs Placebo)** | | | -0.158 | (-0.232,  -0.083) | <0.001 |
| **RMSE** | | 0.067 | | | | **RMSE** | | 0.083 | | | |
| **MAE** | | 0.227 | | | | **MAE** | | 0.333 | | | |

* In the placebo arm

CI, confidence interval; FRR, frequency rate ratio; ICC, intraclass correlation coefficients; MAE, mean absolute error; MMD, monthly migraine day; RMSE, root mean squared error
