# Supplementary material for: Migraine day frequency in migraine prevention: longitudinal modelling approaches
Source: BMC Med Res Methodol. 2019 Jan 23;19:20. doi: 10.1186/s12874-019-0664-5 (PMC6343253; doi:10.1186/s12874-019-0664-5)
Supplement: Supplementary file 4 — Implementation of regression models in Stata. The technical appendix contains the Stata codes which were used to fit the regression models. (DOCX 13 kb) [file 12874_2019_664_MOESM4_ESM.docx]

**Technical Appendix: Implementation of regression models in Stata**

Using the following variables:

freq: number of migraine day episodes in the period

treatment: treatment variable

week: visit week

subjid: patient ID

class: instrumental variable to flag number of events vs number of no events in the period

zeros: variable to flag when zero migraine days

For the multilevel negative binomial regression

the command is as follows:

menbreg freq treatment i.week || subjid:, irr

For the multilevel beta binomial regression

Step 1: this is to follow Guimaraes description of the dataset augmentation:

expand 2

bysort subjid week: gen class=_n-1

replace freq=28-freq if class==0

gen week_cov=week*(class==1)

gen trt_cov=treatment*(class==1)

Step 2: fit a fixed effect negative binomial to run a beta binomial regression

xtnbreg freq class trt_cov i.week_cov, i(subjid) fe

For the multilevel Poisson regression

mepoisson freq treatment i.weeks || subjid:, irr

For the Zero Inflated Negative Binomial (robust standard errors at patient level)

gen zeros=1*(freq==0)

zinb freq treatment i.week, vce(cluster subjid) inflate(zeros)
